# Optical Coupling Efficiency of a Coupler with Double-Combined Collimating Lenses and Thermally Expanded Core Fibers

**DOI:** 10.3390/mi13020324

**Published:** 2022-02-18

**Authors:** Qi He, Zhengang Zhao, Xiaoda Ye, Chuan Luo, Dacheng Zhang, Sifei Wang, Xiaoping Xu

**Affiliations:** 1 Faculty of Information Engineering and Automation, Kunming University of Science and Technology, Kunming 650500, China; hq256_mxr@163.com (Q.H.); luochuan@kust.edu.cn (C.L.); dacheng.zhang@kust.edu.cn (D.Z.); SifeiWang11@163.com (S.W.); xuxiaoping686@163.com (X.X.); 2 Yunnan Key Laboratory of Computer Technologies Application, Kunming University of Science and Technology, Kunming 650500, China; 3 Yunnan Xinyao Semiconductor Materials Co., Ltd., Kunming 650503, China; yexiaoda@sino-ge.com

**Keywords:** fiber optic rotary joint, coupler, thermally-diffused expanded core fiber (TECF), double-combined collimating lens (DCL), coupling efficiency, coupling mismatch fiber

## Abstract

Improving the coupling efficiency of two optical signals is a hot issue, where the efficiency of optical coupling has a significant effect on the signal transmission over the fiber link. To this end, the Large-Beam Fiber Coupler (LBFC) with a Double-combined Collimating Lens (DCL) and a single-mode TEC fiber structure are proposed in this study. Based on the propagation principle of Gaussian beams and the coupling requirements, the coupling mechanism of the fiber coupler and the coupling mismatch between the coupler is analytically modeled. The model and the optical path are optimized, then the ray tracing is used to calculate the coupling efficiency of inter-coupler signals for different SMF. The coupling efficiency is evaluated through experiments in terms of coupling efficiency and the radial, axial, and angular mismatches between the couplers. The results showed that with a large Mode Field Diameter (MFD), better coupling efficiency can be obtained, i.e., a large MFD of 28 μm is tested with its maximal efficiency of 95.16%. Moreover, the angular mismatch has the most significant impact on the coupling efficiency, while the axial mismatch has the least. The use of large MFD can alleviate the angular mismatch and thus improve the optical coupling efficiency.

## 1. Introduction

There is increasing demand for Fiber Optic Rotary Joint (FORJ) for efficient transmission of optical signals from a rotating (stationary) port to a stationary (rotating) port in a wide range of fiber communication applications [1,2], such as military, medical, industrial, and energy applications [3,4,5,6,7]. At present, a passive, coaxial rotary connector using SMF has been extensively adopted owing to its stable performance [7,8]. However, the data carrier of the FORJ is the optical fiber, and there are difficulties in avoiding coupling mismatches caused by assembly errors, vibrations, and other factors during assembly and operation [9]. Moreover, optical signals are transmitted along the optical axis between rotating components, and the axis mismatch will result in the system being unable to transfer the signal. A coupler is the core component of the FORJ signal transmission. Because the beam output by the coupler has a divergence angle, and the core diameter of the common SMF is small. Therefore, coupling optical signals fully into SMF is difficult [10]. A multimode fiber is only suitable for short-range and small-capacity communication due to the large bandwidth, large dispersion, and high loss. As such, investigating the signal coupling of the large-mode field SMF coupler and the coupling mismatch is of considerable significance for improving the optical coupling efficiency of the FORJ.

In 2003, using a Graded-index Lens (G-Lens) collimator, Wencai Jing et al. [8] designed a single-channel optical rotary joint, the insertion loss of 3 dB is obtained experimentally, and the structure relaxes the requirement of assembly accuracy. In 2009, Blomqvist et al. [11] explored a spread-beam fiber-optic coupling system with a single lens and analyzed the effect of different misalignments on coupling efficiency. As Thermally-diffused Expanded Core Fiber (TECF) technology became more widespread, in 2009, George S. Kliros et al. [10] investigated HNA-TEC fibers and SMF-28 fibers and found that the former had lower coupling losses and larger tolerances. In 2012, HuaiXi Chen et al. [12] investigated TECFs to improve the coupling efficiency of waveguide fibers, and the results revealed that the mode field radius expansion of TECFs was more efficient than that of ordinary SMF. However, G-LENS collimators are considerably sensitive to angular mismatch, which creates difficulties for couplers that use conventional SMFs [8]. In 2011, Lei Mi et al. [7] designed an SMF rotary connector using TECF, which was less sensitive to angular tilt than ordinary SMF. In 2020, Yixing Yan et al. [13] analyzed the influence of the positioning error of Fast Axis Collimators (FAC) and Slow Axis Collimators (SAC) on the coupling efficiency, and the positioning error of FAC had the greatest impact on the efficiency. An increasing amount of academics are using ZEMAX software to simulate couplers, reducing the cost of research. In 2019, Mingqian Zhang et al. [14] proposed a coherent fiber array coupler to extend more channels and implemented the simulation with ZEMAX. However, imaging of a single lens has wide aberrations and is prone to divergence. In 2021, Sara Mashalchi et al. [15] designed an angiography system with dual-lens and optimized the system through ZEMAX, the results show that the dual-lens can effectively reduce chromatic aberration and improve image resolution. The studies on the effects of optical fibers and couplers on coupling performance of the FORJ are given in Table 1. To summarize, TECF can improve the coupling efficiency of a coupler, while dual lenses can better correct aberrations and improve efficiency compared with single lenses. Thus, couplers made with large-mode field SMFs combined with dual-combined lenses can be effective in improving the coupling efficiency of the FORJ.

In the present study, a symmetrical fiber coupler with DCLs and TECF was designed. The optical coupling performance of the coupler was analyzed by investigating the structural characteristics of DCLs, the coupling mechanism, the TECF properties, and the coupling mismatch between the couplers. An optical platform was built to test the coupling performance between couplers with six Mode-Field Diameters (MFD) of SMFs. The coupling mismatch was also analyzed to determine the structural parameters of the DCL and TECF in the designed coupler, ultimately improving the optical coupling efficiency.

## 2. Analysis of the Characteristics for Fiber Coupler

### 2.1. Structure Analysis

In practice, the imaging of optical systems is imperfect, and light passing through the optical system creates aberrations that cause defects such as blurring, diffuse spots, and distortion in the image. As seen from a photoreceptor or CCD camera [16], a fiber optic coupler will cause the emitted beam to be parallel when the working distance is close. However, with increases in the working distance, there will be an outward divergence of directional light, increases in the radius of the diffuse spot, and the energy will become progressively weaker. The small core diameter of ordinary SMFs renders difficulties in terms of the coupling of diverging beams, which will lead to a decrease in coupling efficiency. Such effects can be attributed to the small diameter of the collimated spot of the coupler, the small confocal parameter, and the limited working distance. Thus, to design a coupler with a longer working distance, the collimated spot must be increased, which can be achieved by changing the radius of curvature of the lens or by using a DCL [17].

**Figure 1:**
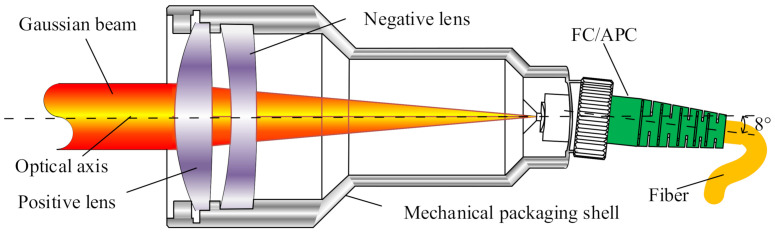
Structure of the large-beam fiber coupler.

As shown by related studies, a DCL with a positive lens and a negative lens will effectively reduce the aberration of the system [18] and expand the collimated beam, while the working distance will have less influence on the coupling. The FC/APC interface was used to facilitate the connection of fibers and reduce losses, and the structure of the fiber coupler is shown in Figure 1. The laser beam was collimated and expanded by a fiber coupler and then converged at the FC/APC interface by another end, and SMF was used to couple the optical signal. Based on the principle of beam expansion and aggregation of couplers, a symmetrical single-channel fiber coupling system was designed for bi-directional signal transmission.

The fiber coupler had no moving parts, was compact, and had a large beam with a narrow divergence angle after collimation. As a result, said fiber coupler was less susceptible to misalignment during adjustment and was highly stable. The FC/APC interface of the coupler was made to be inclined at 8${}^{\circ }$, so as to allow the beam to be directed into the coupler from the vertical focal plane, thereby preventing reflected light from entering the fiber and interfering with the optical signal, and ultimately improving the return loss. The parameters of the coupler are shown in Table 2.

### 2.2. Characteristic Analysis of the TECF

The Gaussian beam from a large beam coupler has a wide spot and a certain dispersion angle, while the core diameter of an ordinary SMF is only 8–10 μm. When such fiber is used to couple the output signal of a coupler, and in the same transmission mode, only part of the optical signal can enter the fiber, which requires high optical alignment accuracy. Due to the large bandwidth, large dispersion, and high loss thereof, the popular multimode fiber with a core diameter of 50 μm is only suitable for short-distance and small-capacity optical communication systems. In summary, to reduce the requirements for optical alignment accuracy and improve coupling efficiency, an SMF of a large MFD was used to couple the optical signal output from the coupler.

**Figure 2:**
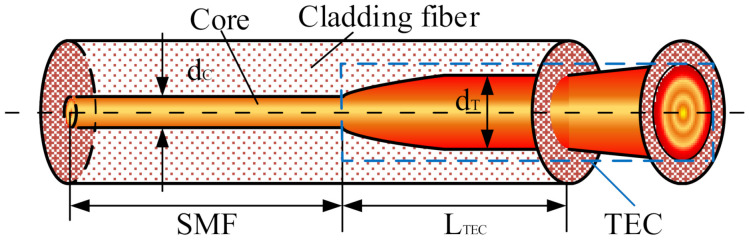
The structure of TECF.

TECF is a specialized SMF with an MFD of 10–30 μm. The Ge atoms contained in the GeO${}_{2}$ dopant in ordinary SiO${}_{2}$ fabric were diffused by heat treatment, resulting in a thermally expanded fabric with a fixed length at one end [19,20]. The structure of TECF is shown in Figure 2. The heat-treated expanded core fiber is highlighted by the blue box and had a larger MFD compared with the SMF part.

A normalized frequency of fiber determined the number of modes within the fiber, as calculated by Equation (1)

(1) $$V=\frac{2\pi rNA}{\lambda }=\frac{2\pi a}{\lambda }\sqrt{n_{1}^{2}-n_{2}^{2}}$$

where *r* is the radius of the fiber. These parameters of the SMF are shown in Table 3. The fiber is heated to make TECFs, and V=2.05 was obtained by substituting these parameters of Table 3 into Equation (1).

When the type of SMF is determined, the normalized frequency is a constant [21]. TECFs always satisfy *V* ≤ 2.405 during heat treatment, such that optical field distribution in TECF is the same as in SMF (fundamental mode distribution), and both transmit as single-mode [22]. The difference between TECF and the ordinary SMF lies in the core diameter. Hence, transmission characteristics of the TECF were analyzed based on Gaussian beams [23], and the relationship between the MFD *D*${}_{T}$ and the core diameter *d*${}_{T}$ of TECFs could be denoted as:

(2) $$D_{T}=\frac{d_{T}}{\sqrt{\ln V}}$$

The six SMFs with different MFD were made from heated SMF, with 10 μm and 11 μm as normal SMFs and 14 μm, 17 μm, 20 μm, and 28 μm as TECFs. To meet the efficient coupling of large beam fiber coupling systems, the fiber ports were equipped with FC/APC connectors.

### 2.3. Analysis of the Coupling Mechanism

**Figure 3:**
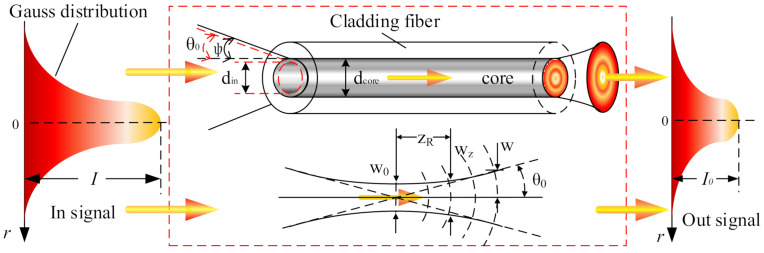
Diagram of how the fiber optic meets the signal coupling requirements.

Coupling efficiency is a significant parameter for measuring the performance of fiber couplers. Before analysis, determining whether the fiber can meet coupling requirements of the output signal of the coupler is necessary [24]. Figure 3 shows a schematic diagram of a fiber meeting the coupling requirements.

The output beam diameter of the FORJ is *d*${}_{in}$ (the diameter of dispersion spot after imaging), and the fiber core diameter is *d*${}_{core}$. To meet Requirement 1, the beam diameter must be less than or equal to the fiber core diameter, and the beam can be fully coupled into the fiber only if the divergence angle θ of the beam incident on the fiber end face is less than 2φ, which is calculated by Equation (3)

(3) $$\left\{\begin{array}{c} d_{in}\leq d_{core}\\ \theta =\frac{\lambda }{\pi \omega_{0}}\leq 2\varphi \end{array}\right.$$

where λ is the wavelength of light and $\omega_{0}$ is the Gaussian beam waist radius, φ=arcsin(NA), $NA=\sqrt{n_{1}^{2}-n_{2}^{2}}$, NA is the numerical aperture of the fiber, *n*${}_{1}$ is the core refractive index, and n${}_{2}$ is the cladding refractive index.

The laser beam is distributed in the SMF as a fundamental mode Gaussian function. As such, in the coupler in which pigtail is an SMF, the coupling efficiency is approximately calculated according to the Gaussian beam [25]. The coupling efficiency is an effective measure of the signal transmission performance of a coupler. When the coupling efficiency is higher, the signal loss will become smaller [26]. The coupling efficiency of the SMF was calculated by

(4) $$\eta =\frac{|\;\int \int \;E_{1}E_{2}{d\sigma |}^{2}}{\left[\;\int \int \;\right|E_{1}{|}^{2}d\sigma ][\;\int \int \;|E_{2}{|}^{2}d\sigma ]}$$

where *E*_1_ is the fundamental mode distribution function of the transmitting fiber and *E*_2_ is the fundamental mode distribution function of the receiving fiber. The Gaussian beam could be denoted as

(5) $$E\left(x,y\right)=A_{0}\exp\left[-\frac{x^{2}+y^{2}}{\omega_{0}^{2}}\right]$$

where A${}_{0}$ is the amplitude constant, and the coupling efficiency $\eta_{0}$ of an SMF coupler is the ratio of the output power *P*(*O*) of the beam to the input power *P*(*i*), which is the superposition of the mode field of the Gaussian beam between the coupler. By combining Equation (5) and Equation (4), the coupling efficiency of an SMF coupler could be obtained as denoted in Equation (6)

(6) $$\eta_{0}=\frac{P(O)}{P(i)}={\left|A_{1}A_{2}\;\int \int \;\exp\left[-\frac{x^{2}+y^{2}}{\omega_{1}^{2}}\right]\exp\left[-\frac{{\left(x+X\right)}^{2}+y^{2}}{\omega_{2}^{2}}\right]d\sigma \right|}^{2}$$

where $\omega_{1}$ and $\omega_{2}$ are the beam radii of the two couplers and *X* is the mismatch between the coupler. The coupling efficiency $\eta_{0}$ of a large-beam fiber coupler versus the coupling loss is expressed in Equation (7)

(7) $$Loss=-10\log_{10}\eta_{0}$$

### 2.4. Coupling Efficiency of the LBFC

The fiber coupler is a core component of the FORJ and consists of two symmetrical lenses, a collimating lens, and a converging lens. The collimating lens expands and collimates the beam, and the converging lens converges the collimated beam into the fiber [27], thereby reducing the requirement for mechanical alignment accuracy therebetween. Common collimating lenses include G-Lens, conventional lenses (C-Lens), aspherical lenses, and combination lenses, of which the C-Lens is used more often owing to the low cost and stable performance thereof [28]. In the present study, a single channel large beam fiber coupler was designed based on combined lenses, and the coupling efficiency was analyzed.

**Figure 4:**
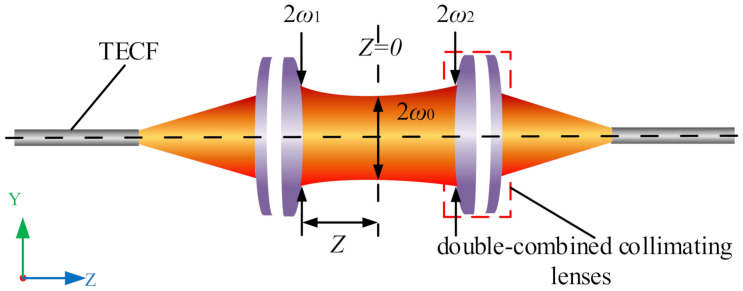
The LBFC exactly coupled.

The LBFC collimates and expands the incident light into a nearly parallel beam with a radius of $\omega_{1}$, and the theoretical approximation could be calculated using Equation (8)

(8) $$\omega_{1}\approx 2\lambda \left(\frac{f}{\pi D_{T}}\right)$$

where *f* is the focal length of the coupler. When the mode field of the fiber coupler is perfectly matched ($\omega_{1}$ and $\omega_{2}$ have the same distribution) and satisfies Equation (3), the optical signal is coupled exactly into the fiber, as shown in Figure 4.

The light intensity at the Gaussian beam waist position decreases along the z-axis as a Gaussian function from the center outwards, and the beam width ω expands outwards with the y-coordinate according to the hyperbolic law, the size of the beam at any *z* ω(z) could be calculated using Equation (9)

(9) $$\omega \left(z\right)=\omega_{0}\sqrt{1+{\left(\frac{z}{Z_{0}}\right)}^{2}}$$

where *Z*${}_{0}$ is the Rayleigh length, which is calculated using Equation (10)

(10) $$Z_{0}=\frac{\pi \omega_{0}^{2}}{\lambda }$$

It is known from Equation (9) that ω(z) is related to *z* and *Z*${}_{0}$. The Gaussian beam in the range of *z* ≤ *Z*${}_{0}$ is collimated into nearly parallel light, and the larger the *Z*${}_{0}$, the larger the collimation range and the smaller the divergence angle. As shown by Equation (9), ω(z) is related to *z* and *Z*${}_{0}$. The Gaussian beam in the range of *z* ≤ *Z*${}_{0}$ is collimated into nearly parallel light, and the larger the *Z*${}_{0}$, the larger the collimation range and the smaller the divergence angle. Combining Equations (9) and (10) yields

(11) $$\omega {\left(z\right)}^{2}\omega_{0}^{2}=\omega_{0}^{4}{\left(\frac{\lambda z}{\pi }\right)}^{2}$$

When *z* is at a certain length, the value of the rightmost term of Equation (11) is considerably small, which can be approximated to 0. As such, the bundle width ω(z) of the fiber coupler approximates the bundle waist $\omega_{0}$ at *z*, that is, $\omega_{0}$, $\omega_{1}$, and $\omega_{2}$ are equally distributed. Therefore, the coupling efficiency could be calculated using $\omega_{1}$.

**Figure 5:**
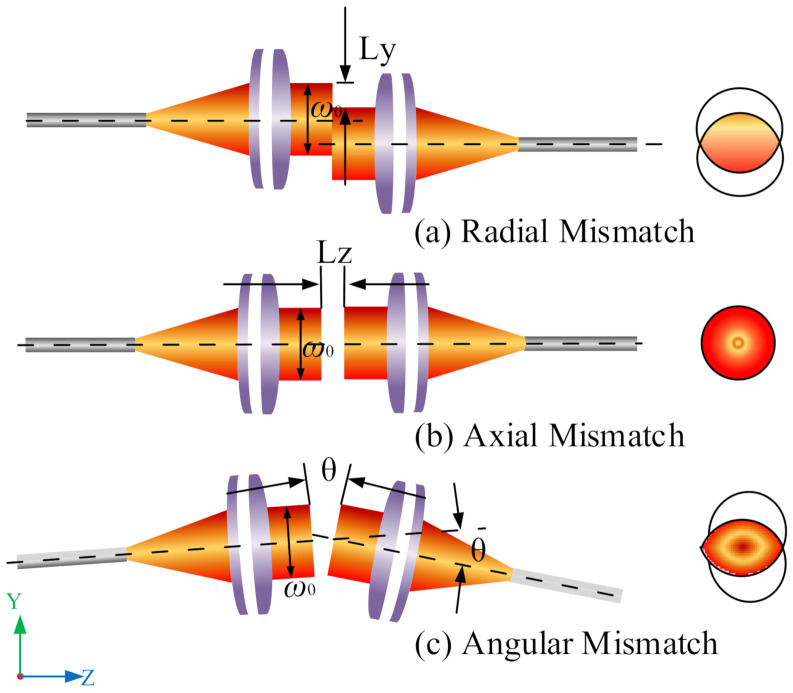
Coupling mismatch of coupler.

The coupler was mounted on the precision shaft for calibration. The rotation of the spindle would produce mechanical vibrations of different dimensions and other factors, which would make the optical axis eccentric. Three types of mismatches may occur between couplers [29], namely radial mismatch (*L*${}_{y}$), axial mismatch (*L*${}_{z}$), and angular mismatch (θ), as shown in Figure 5.

The mode field distribution of the fiber coupler is consistent. The coupling efficiencies caused by three kinds of mismatches were calculated based on the beam waist of the coupler and Equation (6) [30], where the coupling efficiency $\eta_{L_{y}}$ caused by radial mismatch could be calculated by Equation (12)

(12) $$\eta_{L_{y}}=\exp\left(-\frac{L_{y}^{2}}{\omega_{1}^{2}}\right)$$

where *L*${}_{y}$ is the radial mismatch, which is the distance between the axis of two symmetric lenses.

The coupling efficiency $\eta_{L_{z}}$ resulting from the axial mismatch is calculated using Equation (13)

(13) $$\eta_{L_{z}}=\frac{4}{4+{\left(\frac{\lambda L_{z}}{\pi \omega_{1}^{2}}\right)}^{2}}$$

where *L*${}_{z}$ is the axial mismatch, which is the distance perpendicular to the axis of the two lenses.

The coupling efficiency $\eta_{\theta }$ resulting from the angular mismatch is calculated by Equation (14)

(14) $$\eta_{\theta }=\exp\left[-{\left(\frac{\pi \omega_{1}\tan\theta }{\lambda }\right)}^{2}\right]$$

where θ is the angular mismatch, which is the angle between the axis of the two lenses. When the coupling efficiency of the three kinds of mismatches is superimposed, the coupling efficiency $\eta_{0}$ between the couplers could be calculated by Equation (15)

(15) $$\eta_{0}=\eta_{L_{y}}\eta_{L_{z}}\eta_{\theta }$$

**Figure 6:**
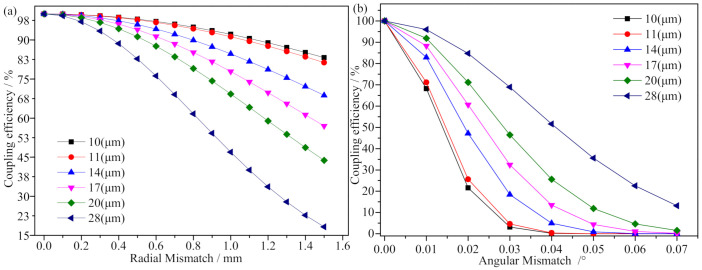
Coupling mismatch curves for fiber couplers with different MFD. (**a**) Radial mismatch, (**b**) angular mismatch.

From Equation (15), when the fiber coupler satisfies the coupling condition without mismatch, the coupling efficiency is $\eta_{0}=100\%$. The coupling efficiency of the radial mismatch and angular mismatch present when receiving the coupler output optical signal from SMFs with different MFDs were calculated, as shown in Figure 6.

As shown in Figure 6a, the coupling efficiency decreased as the MFD and radial mismatch of the fiber increased. Compared with ordinary SMFs, large mode field fibers require higher radial mismatch. When the mismatch reaches 1.5 mm, the coupling efficiency is less than 20% for fibers with an MFD of 28 μm and over 85% for 10 μm. The coupling performance of fibers with small SMFs was more stable when the coupler produced a radial mismatch of millimeters, which can be controlled in practice.

As shown in Figure 6b, as the angular mismatch increases, the coupling efficiency gradually decreases. In the case of the same angular mismatch, the larger the MFD, the higher the efficiency, and the angular mismatch increases the coupling efficiency slowly decreases. The FORJ operation is subject to vibration and other effects, which render difficulties in controlling angular mismatch due to the high angular requirements. Thus, large mode field fibers can be used to improve the effect of angular mismatch on coupling efficiency.

The working distance of the coupler in the present study was long, and the requirement for axial distance was low. The results were calculated from Equation (13) to show that the coupling efficiency varied in the range of 0–0.00001% with increases in *L*${}_{z}$ and decreases in $\omega_{1}$. As such, the coupling effect of the axial distance aligner was small and could be ignored.

## 3. Optimization Analysis for Simulation

### 3.1. Modeling

To analyze the effect of fiber MFD on coupler coupling efficiency, the coupling between the couplers was simulated by ZEMAX using a ray-tracing method. The large beam coupler was modeled in Lens Data Editor by building sequential DCLs. The numerical aperture of the coupler was set to 0.14 according to the parameters in Table 2, and the lens surface type is standard. The working distance between the DCL was set to 0–100 mm, the distance *f* from the light source to the lens was set to 37 mm, and the types were all variable (V). Moreover, the light source is set as a divergent point light source with a maximum divergence angle of 8${}^{\circ }$ and a wavelength of 1550 nm. The lens model parameters, system optical path, and imaging were optimized by adding optimization functions and operands after adjusting the basic parameters such as radius of curvature, focal length, the field of view, and diaphragm surface for the lens.

**Figure 7:**
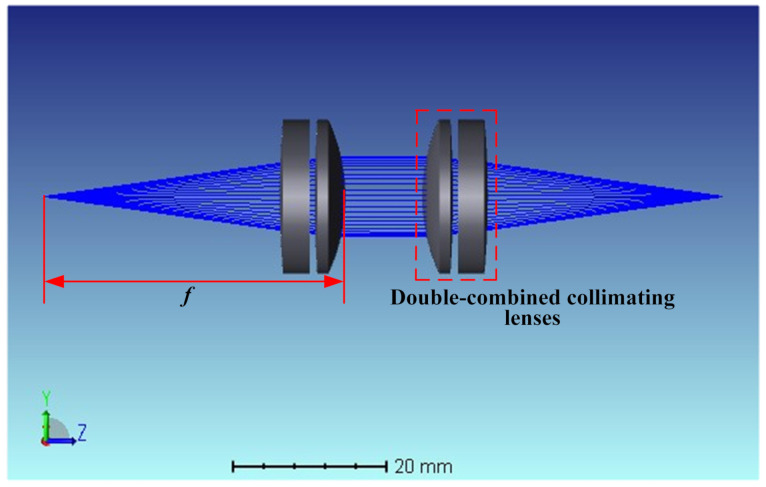
Coupling mismatch for coupler.

The system model and optical path are shown in Figure 7, in which the structure of DCL is symmetrical, which realizes beam expansion, collimation, and aggregation of the optical path. The diameter of the collimated beam is larger, and the increased working distance has little effect on the divergence of the spot.

Table 4 shows the parameters of the optimized DCL model, where the focal length *f* is fixed.

### 3.2. Analysis of Models

**Figure 8:**
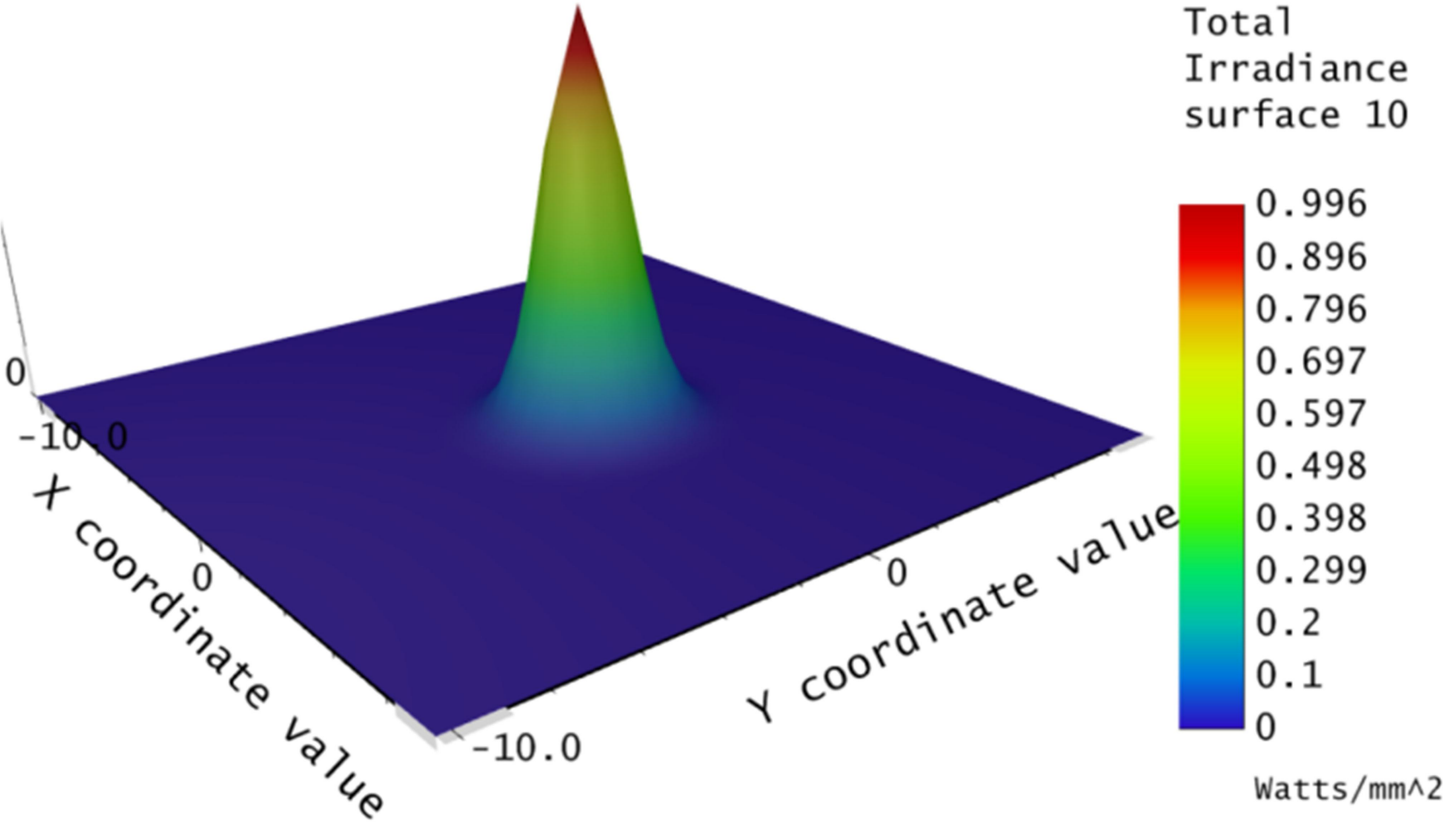
Three-dimensional energy distribution of the coupler.

The three-dimensional energy distribution of the beam after passing through the coupler was obtained by means of Physical Optical Propagation (POP), as shown in Figure 8. The beam energy distribution in the figure was Gaussian, with the strongest energy distribution at the center after the beam had passed through the DCL. Therefore, when designing the coupling experiment, the central optical axes of the two DCLs had to be adjusted to the same line, and the beam waist radius was used to calculate the coupling efficiency.

The RMS Radius, AIRY Radius, and GEO Radius values were obtained from the Spot Diagram (SD) after imaging of the coupler. The SD is an image of the light emitted from a light source that is not concentrated at one point due to aberrations in the optical system, resulting in a circular diffuse spot with a certain radius. If the value is smaller, the imaging quality of the coupler will be better, and the energy of the beam will be more concentrated. Therefore, the imaging quality of the coupler was determined from the value of SD in the present study.

**Figure 9:**
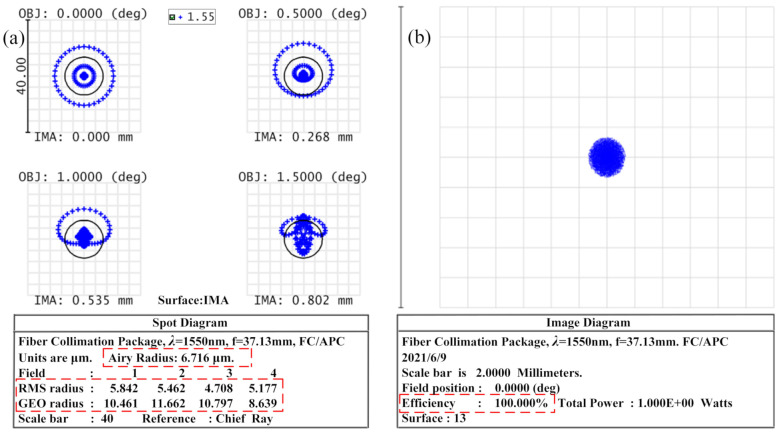
Image quality evaluation chart. (**a**) Spot diagram, (**b**) geometric image analysis.

Figure 9 shows the image quality evaluation of the coupler, in which Figure 9a is the SD of four different fields of view. The RMS Radius of the 0 fields of view was 5.842 μm, the AIRY Radius was 6.761 μm, and the GEO Radius was 10.461 μm. The radius of the diffuse spot was about 7 μm, at which time the RMS was smaller than the AIRY value to reach the diffraction limit. As such, if the optical signal was to be fully coupled into the fiber, the radius of the fiber had to be greater than the radius of the dispersion spot. To determine whether the MFD of the fiber met the coupling conditions, the size of the dispersion spot was used.

The method of Geometric Image Analysis (GIA) was used to calculate the efficiency of the point object coupling to the fiber, which could be used to visualize the shape of the imaged object, as shown in Figure 9b.

### 3.3. Coupling Analysis

The diffuse spot diameter after coupler imaging was greater than 14 μm, while the MFD of an ordinary SMF was about 10–12 μm, which was less than 14 μm and did not meet the coupling requirements. Therefore, fibers with an MFD greater than 14 μm were required to couple the output optical signal from the coupler.

**Figure 10:**
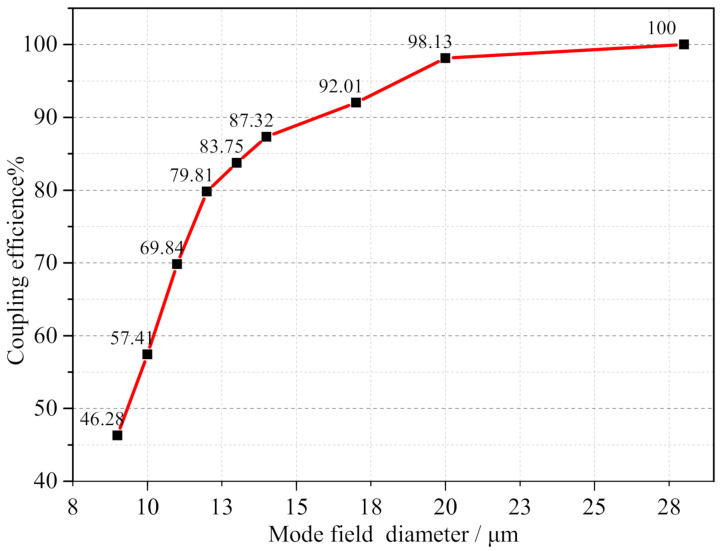
Coupling efficiency curves under different MFD.

The non-sequential method was adopted, which allows for the coupling of optical signals from the SMF analog coupler array with different MFDs to be realized. To build the fiber coupler, the SMF was added after the two DCLs. The coupling efficiency of the large-beam fiber coupler was obtained by GIA, as shown in Figure 10.

An observation can be made from Figure 10 that in the absence of mismatch, as the MFD of the SMF increased, the coupling efficiency of the fiber coupler gradually increased. When the MFD of fiber increased from 10 μm to 28 μm, the coupling efficiency also gradually increased. However, when the MFD increased to 16 μm, the increase in efficiency became smaller. Here, the spot size output by the coupler was basically the same as the fiber diameter, and coupling condition Equation (3) was satisfied so that the optical signal could be coupled to the fiber. When the MFD increased to 28 μm, the coupling efficiency reached 100%. Thus, the use of a large MFD could significantly improve the coupling efficiency.

## 4. Experimental Analysis

### 4.1. Experiment Build

When designing optical experiments, in addition to the problems caused by the superposition and coupling mismatch of the optical field of the fiber coupler, the loss caused by the rotation of the FORJ also needs to be analyzed. As such, according to the coupling requirements of the fiber coupler and the results of the coupling mismatch analysis, as well as the results of simulation for optical devices such as coupler with ZEMAX, an optical coupling experimental system was conducted using an optical platform. The optical coupling loss of the fiber coupler was analyzed experimentally to ensure that the optical axes between the couplers were coincident, thereby providing a significant basis for the design of the mechanical structure of the large beam FORJ.

**Figure 11:**
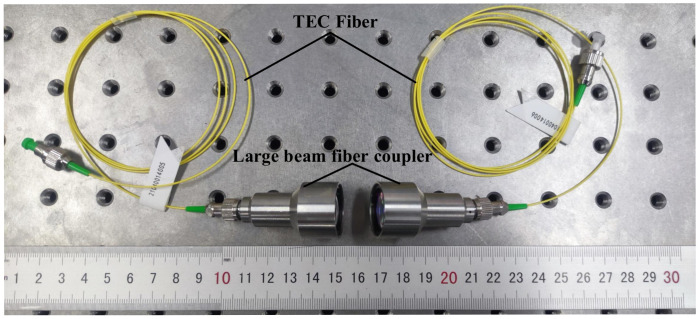
Large-beam TECF coupler.

For the present experiment, a high degree of precision in optical instrumentation was required. The main optical components are a large beam coupler, a precision air-bearing vibration isolation optical stage, a high precision multi-axis displacement stage, a laser light source, a high precision power meter, and six SMFs with different MFDs. Figure 11 shows the optical fiber coupler designed in the present experiment, which consisted of two identical couplers and two identical TECFs with FC/APC connectors. The optical fiber coupler was fixed on the multi-axis translation stage during calibration.

**Figure 12:**
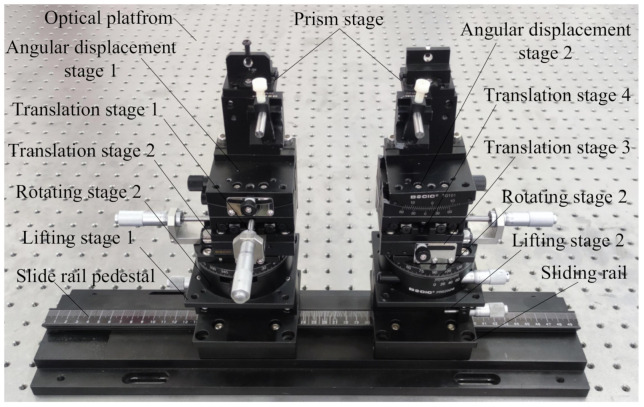
Precision multi-axis displacement table.

The multi-axis displacement stage used in the present experiment was accurate to 0.001 mm and 0.01${}^{\circ }$, and consisted of four translation stages, two rotation stages, two angular displacement stages, two elevation stages, two precision prism stages, two slide rails, and one sliding seat, as shown in Figure 12.

**Figure 13:**
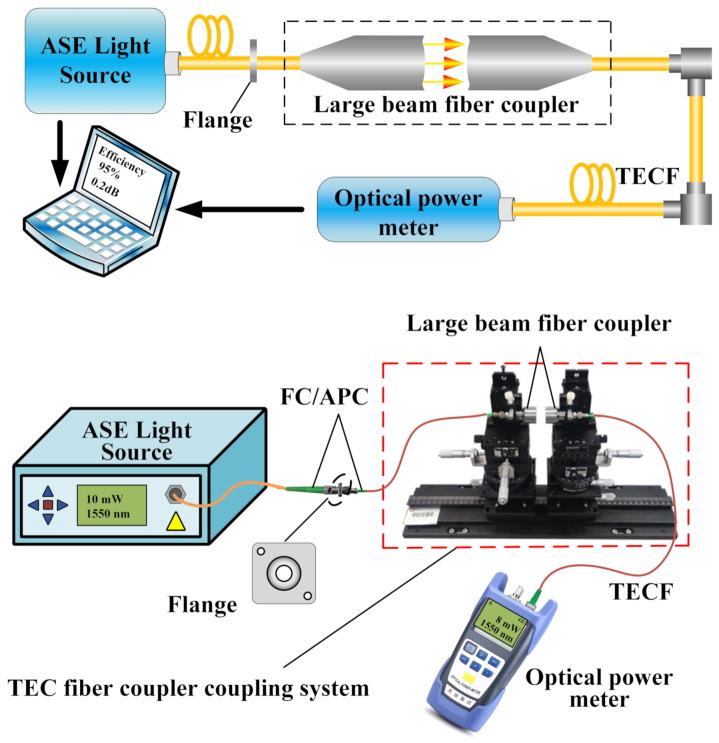
Experimental measurement system of the LBFC.

To obtain the coupling performance of the TECF coupler, an optic platform was built to design the coupling experiments system according to the aforementioned theoretical research and the characteristics of the optical device, as shown in Figure 13. The spectral range of the Amplified Spontaneous Emission (ASE) broadband light source used was 1515–1595 nm and the output power was 10 mW. The resolution of the handheld optical power meter was 0.01 dB, the insertion loss was less than 1.5 dB, and the spectrum thereof was adapted to the ASE light source. The ASE output bandwidth light source was injected into a fiber coupler through a flange, which collimated and expanded the beam and then converged the beam back into the fiber. The power and loss were detected by the optical power meter. During calibration, the fiber coupler was fixed on the precision prism stage, the output power of the fiber coupler was adjusted to the maximum through the multi-axis translation stage, and the working distance was determined. The coupling efficiency was analyzed through the host system.

### 4.2. The Influence of the MFD for Fiber on the Coupling Efficiency

**Figure 14:**
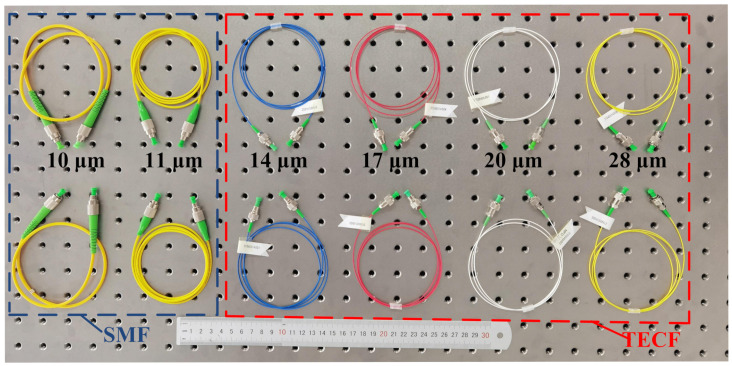
The SMFs with different MFDs.

According to the coupling requirements of the fiber coupler and the size of the dispersion spot obtained by the ZEMAX simulation, the influence of fibers with different MFDs on the coupling efficiency of the coupler was analyzed. The ordinary SMFs with MFDs of 10 μm and 11 μm and TECFs with MFDs of 14 μm, 17 μm, 20 μm, and 28 μm were mainly used to couple the optical signals output from couplers, as shown in Figure 14.

**Figure 15:**
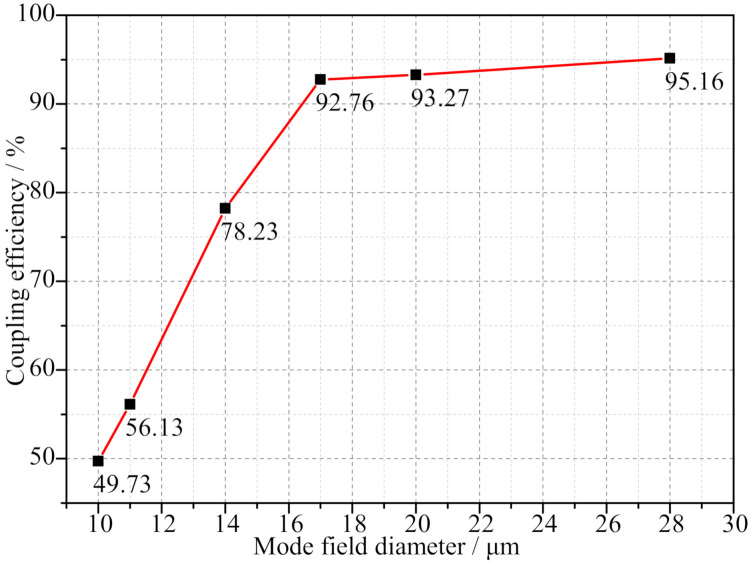
Measured curves of coupling efficiency.

When the wavelength of the light source was 1550 nm and the power was 10 mW, the output power of the fiber coupler was maximized by adjusting the multi-axis translation stage. The coupling efficiency was then obtained by replacing the SMFs with six MFDs through the FC/APC interface of the coupler, as shown in Figure 15.

As shown in Figure 15, the coupling efficiency of the large-beam TECF coupler increased as the MFD of the fiber increased. The coupling efficiency increased more rapidly from 10 μm to 17 μm, with an increase of 43.03%. However, the coupling efficiency increased from 92.76% to 95.16% as the MFD increased from 17 μm to 28 μm, with a more moderate increase in the segment.

In the actual measurement, the fiber coupler is affected by the vibration caused by the adjustment device, and the optical device itself has errors. During the coupling process, the beam still has a small divergence angle θ, and the beam waist radius $\omega_{1}$ and $\omega_{2}$ are not equal. Therefore, the mode fields of the two beams cannot be completely matched, and the coupling requirements cannot be met. Moreover, there is coupling loss, so the coupling efficiency still has a gap with the simulation results, but the gap is small, and the overall coupling performance is excellent. Such results are consistent with the results obtained from the simulations in Section 3.

### 4.3. The Influence of the Mismatch on the Coupling Efficiency

The FORJ is subject to vibration from the external structure during operation, which can cause a mismatch between the couplers and affect the mode field matching. The transitional mismatch will fail to transmit the optical signal, requiring recalibration of the couplers and the design of the FORJ mechanical structure. Hence, to improve the coupling efficiency of optical signals, the mismatch between fiber couplers was analyzed through experiments.

**Figure 16:**
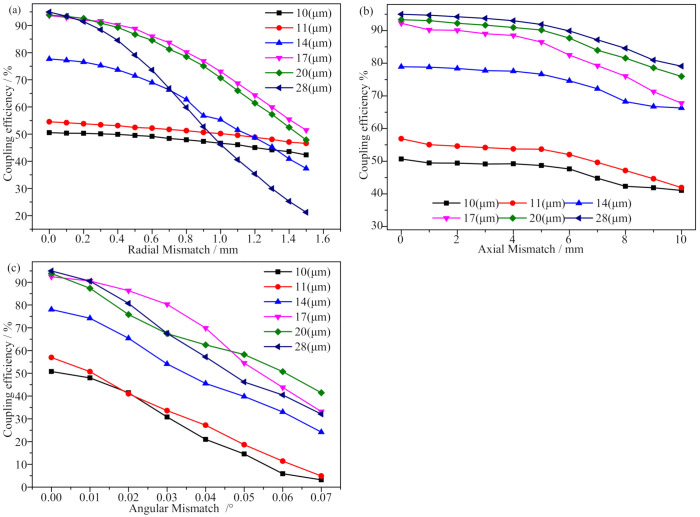
Actually measured coupling curves that produce mismatches. (**a**) Radial mismatch, (**b**) axial mismatch, and (**c**) angular mismatch.

The shock absorption and leveling effect of the optical platform could reduce external interference. The optical axis of the coupler could be adjusted on the same straight line with the help of visible light, and the multi-dimensional fine-tuning could be performed at the translation stage to maximize the output power. Separate adjustment of the multi-axis displacement stage would produce radial mismatch, axial mismatch, and angular mismatch. Finally, an SMF array with six different MFDs was used to couple the output optical signal for the coupler. The experiment was tested in several cycles, and the coupling efficiency curve was plotted as shown in Figure 16.

The coupling efficiency of the large-beam TECF coupler is shown in Figure 16a when radial mismatch was present for an SMF with different MFDs. The larger the MFDs the higher the coupling efficiency between the couplers. As the radial mismatch increased, the image quality of the coupler was poorer, the spot increased, the image was shifted, several signals encountered difficulties coupling to the fiber, and the coupling efficiency exhibited a decreasing trend. The maximum variation of the coupling efficiency was within 70% and the minimum variation was within 7%, the overall coupling efficiency changed more uniformly, but the MFD of 28 μm fiber decayed faster after 0.3 μm, which could be attributed to the large MFD of the fiber and the beam in the fiber mode field matching degree decreasing, resulting in a decrease in the transmission efficiency of the fiber. The beam emitted by the coupler has a certain divergence angle and aberration, which cannot fully satisfy the coupling condition of Equation (3), resulting in a beam diameter much larger than 10 μm. Therefore, only part of the optical signal is coupled into the 10 μm fiber, and the loss is increased, thus making the coupling efficiency too low.

The measured coupling efficiency curve in the presence of axial mismatch for the large-beam TECF coupler can be seen in Figure 16b, which reflects the coupling efficiency of the SMF coupling output signal for different MFDs. As the axial distance between couplers increased, the coupling efficiency decreased gradually, from 0 mm to 10 mm, with an average decrease of 16%. When adjusting the translation stage, due to factors such as uneven force, a relatively small misalignment between the couplers occurred, which caused the coupling efficiency to rapidly decrease compared with the theoretical results. Such findings also reflect that the radial mismatch and angular mismatch had a serious impact on the coupling efficiency.

The measured coupling efficiency curve in the presence of angular mismatch for the large-beam TECF coupler can be seen in Figure 16c, in which angular mismatch had the most significant effect on the coupling efficiency. As the angular mismatch increased from 0${}^{\circ }$ to 0.07${}^{\circ }$, the coupling efficiency decreased by 50% on average. The fiber coupler required high angular accuracy and was difficult to control, but the larger MFD at the same angular mismatch, the higher the coupling efficiency. Thus, using TECFs with larger MFDs would significantly improve the coupling effects of angular mismatch on the fiber coupler.

In summary, in the absence of mismatch, the larger the MFD of the fiber, the higher the coupling efficiency. The use of SMFs with large MFDs would effectively improve the coupling efficiency of the coupler, and the aforementioned experimental results are in general agreement with the results calculated in Section 2 and Section 3. To ensure that the demodulation equipment could collect the optical signal (coupling efficiency over 50%) when all mismatches were present, a TECF with an MFD of 20 μm was selected. The working distance of the coupler was also set to 10 mm, and the radial mismatch was controlled to 0.5 mm, the angular mismatch was controlled to 0.04${}^{\circ }$, and the axial mismatch was controlled to 1 mm, resulting in a coupling efficiency of 50.4% that eventually allowed for bi-directional transmission of optical signals in the fiber coupler to be achieved.

## 5. Conclusions

In the present study, the structure of the single-mode large-beam TECF coupler was analyzed. The coupling mechanism was explored when the pigtail of the fiber coupler had different MFDs, and the three mismatches that were present during the use of the fiber coupler were analyzed in detail. The model for the fiber coupler was established using ZEMAX, and the coupling efficiency was calculated. The critical points of the FORJ coupling method, the characteristics of the DCL, and the coupling mismatch were combined to build an optical experiment platform to verify the theoretical and simulation calculation results, and the following results were obtained:

1. When the fiber coupler met the coupling requirements, the larger the MFD of the fiber, the higher the coupling efficiency. If there was a coupling mismatch between the fiber couplers, the angular mismatch had the most influence, followed by the radial mismatch, with the axial mismatch being the smallest. When the MFD of the fiber was 10 μm, the coupling efficiency of the coupler was 57.41% through simulation, and 49.73% in the experiment. When the MFD was 28 μm, the simulation coupling efficiency was 100%, while the experiment was 95.16%. As such, compared with an ordinary SMF, the coupling efficiency of the large-mode field TECF to the coupler was significantly improved.
2. When all mismatches of the coupler were present, a 20 μm fiber was chosen to couple the signal. The coupling efficiency could reach over 50.4% (loss within 3 dB), which met the signal transmission requirements of the FORJ for a single-mode large-beam TEC. The aforementioned conclusions can guide the design of such fiber optic rotary connector.
3. The double-lens expands and collimates the beam, has a small divergence angle, a large working distance, and stable transmission performance. Moreover, this lens can better reduce the influence of aberration on the coupling efficiency.
4. This work can provide some instructive technical support for the bi-directional transmission of fiber couplers, the online monitoring of signals with fiber grating sensing, and the research on the transmission performance of couplers under rotating conditions.

## Figures and Tables

**Table 1:** Study of coupling performance.

References	Method/Structure	Results
Jing et al. [8]	SMF G-lens	Relaxed the assembly accuracy requirement Achieved an insertion loss of 3 dB
Blomqvist et al. [11]	Single lens	Radial displacement of 3.7 μm reduces the coupling efficiency with 50%
George et al. [10]	TECF SMF	TECF has lower coupling losses Larger tolerances
Chen et al. [12]	TECF	The TECF mode field radius extension is more effective than the SMF
Mi et al. [7]	TECF G-lens	Less sensitive to angular tilt
Yan et al. [13]	FAC SAC	The positioning error of FAC had a greater effect on the coupling efficiency than SAC
Zhang et al. [14]	ZEMAX Coupler	Achieved the simulation of the fiber array coupler
Sara et al. [15]	ZEMAX Dual-lens	Dual-lenses reduce chromatic aberration and improve image resolution

**Table 2:** Parameters of the coupler.

Wavelength/nm	Focal Length/mm	Numerical Aperture	Length/mm	Width/mm
1550	37	0.14	42	25

**Table 3:** These parameters of the SMF.

λ/nm	$n_{1}$	$n_{2}$	$d_{c}/$μm	MFD/μm
1550	1.468	1.462	8.2	10

**Table 4:** Parameters of the model for a large-beam DCL.

Comment	Radius/mm	Thickness/mm	Naterial	Coating
OBJECT	Infinity	0.0	−	−
f	Infinity	31.3	−	−
Lens1	115.6	2.7	N−SF6	THORC
Lens1	43.7	1.8	N−SF6	THORC
Lens2	153.8	4	N−SF6	THORC
Lens2	−23.7	60	N−SF6	THORC

## Data Availability

The data that support the findings of this study are available from the corresponding author upon reasonable request.
